# Dietary supplementation of *Bacillus subtilis* or antibiotics modified intestinal microbiome of weaned pigs under enterotoxigenic *Escherichia coli* infection

**DOI:** 10.3389/fmicb.2022.1064328

**Published:** 2022-12-23

**Authors:** Cynthia Jinno, Xunde Li, Yanhong Liu

**Affiliations:** ^1^Department of Animal Science, University of California, Davis, Davis, CA, United States; ^2^Department of Population Health and Reproduction, School of Veterinary Medicine, University of California, Davis, Davis, CA, United States

**Keywords:** antibiotics, *Bacillus subtilis*, *Escherichia coli* challenge, microbiome, weaned pigs

## Abstract

Our previous research reported that supplementation of *Bacillus subtilis* DSM 25841 promoted growth and disease resistance of weaned pigs under enterotoxigenic *Escherichia coli* (ETEC) challenge and its efficacy is comparable to carbadox. This follow-up study aimed to characterize the effects of ETEC infection, supplementing *B. subtilis* DSM 25841 or carbadox on intestinal microbiota of pigs. Forty-eight weaned pigs (6.17 ± 0.36 kg BW) were randomly allotted to one of four treatments: negative control (NC), positive control (PC), antibiotics (AGP, 50 mg/kg of carbadox), and direct fed microbials (DFM, 2.56 × 10^9^  CFU/kg of *B. subtilis*). The experiment lasted 28 days with 7 days before and 21 days after first *E. coli* inoculation (day 0). Pigs in the PC, AGP, and DFM groups were orally inoculated with F18 ETEC for 3 consecutive days with 10^10^  CFU per dose per day. Fecal samples were collected on day −7, and day 7 and day 21 post inoculation, digesta samples were collected from jejunum, ileum, and distal colon on day 21 post inoculation to perform 16S rRNA sequencing. Sampling days and locations influenced (*p* < 0.05) Chao1 index and beta-diversity. Age increased (*p*  < 0.05) the relative abundance of Firmicutes but decreased (*p* < 0.05) the relative abundance of Bacteroidetes in feces. ETEC infection increased (*p*  < 0.05) the relative abundance of Proteobacteria in feces on day 7 post inoculation. AGP reduced (*p* < 0.05) relative abundance of Firmicutes and *Lactobacillaceae* in feces compared with PC and DFM. AGP reduced (*p* < 0.05) relative abundance of *Bifidobacteriaceae* in jejunum and ileum, while DFM reduced (*p* < 0.05) relative abundance of *Actinomycetaceae* in jejunum and *Lachnospiraceae* in ileum, compared with PC. Pigs fed with DFM had greater (*p* < 0.05) relative abundance of *Ruminococcaceae*, *Veillonellaceae*, *Bifidobacteriaceae* in jejunum, *Lactobacillaceae* in ileum and colon, and *Bifidobacteriaceae* in colon than pigs in AGP. Current results indicate that carbadox or *B. subtilis* had stronger influences on microbial diversity and composition in ileum than other intestinal segments and feces. Supplementation of *B. subtilis* could increase or maintain the relative abundance of beneficial bacteria in ileum compared with carbadox.

## Introduction

Transitioning from farrowing to nursery stage is known to aggravate the stress of pigs raised for pork production. Newly weaned pigs can experience extreme discomfort when separated from their sows, as they are accompanied by a sudden change in diet, environment, and social life conditions (Campbell et al., 2013). Prolonged exposure to stress has shown to adversely impact the health and performance of pigs, resulting in huge economical losses. Stress can also induce microbial imbalance in the gut and increase vulnerability to pathogens (Madison and Kiecolt-Glaser, 2019). Enterotoxigenic *Escherichia coli* (ETEC) is an intestinal pathogenic strain that is commonly known to induce secretory diarrhea and intestinal inflammation in pigs under post-weaning stress (Lallès et al., 2004; Fairbrother et al., 2006). In-feed antibiotics, commonly known as antibiotics growth promoters (AGP), were used to apply into nursery diet to alleviate post-weaning diarrhea and to promote growth in weaned pigs (USDA, 2007). On globally estimation, pigs on average consumed approximately 172 mg of antimicrobials per kilogram body weight globally, which was greater than the amounts that cattle and chicken consumed (Boeckel et al., 2015). The global antimicrobial consumption is estimated to increase by 67% between 2010 and 2030 (Boeckel et al., 2015). The extensive use of AGP, however, may increase the chance of dispersing antimicrobial residues and the development of antimicrobial resistance in bacteria (Menkem et al., 2019; Ma et al., 2021). In addition, AGP can modify the gut microbiota and essentially kill beneficial bacteria, thus, may increase the susceptibility to other infections in weaned pigs (Looft et al., 2014). The heightening concerns toward antibiotics and AGP uses have led to the prohibited use of AGP (FDA-2011-D-0889) in livestock for growth-promoting purposes (FDA, 2013). Thus, alternatives to in-feed antibiotics are to be sought immediately.

The importance of gut microbiome in human and animal health, particularly in gut health has been largely reviewed (Kamada et al., 2013; Fouhse et al., 2016; Ke et al., 2019). During weaning, pigs were reported to have temporary loss of microbial diversity in their intestinal tracts, including a decrease in *Lactobacillus* spp. and an increase in a family of pathogenic bacteria such as *Enterobacteriaceae* (Cao et al., 2016; Gresse et al., 2017; Tian et al., 2017). The disturbance of gut microbiome, in combination with immature intestinal immunity, can lead to the expansion of enteric pathogens, such as ETEC infection. Direct-fed microbials (DFM) have been applied to nursery diets to enhance intestinal health, disease resistance, and performance (Liao and Nyachoti, 2017). DFM are categorized into three main groups, including lactic acid-producing bacteria, yeast, and *Bacillus* spp. Compared with other categories, *Bacillus*-based DFMs are spore-forming and thermostable, thus, easy to handle for feed storage and processing. Dietary supplementation of *Bacillus subtilis* was reported to enhance growth performance and diarrhea in nursery pigs by modulating the intestinal immunity and microbiota (Hu et al., 2014; Luise et al., 2019). In our previous studies, we observed that supplementing *B. subtilis* DSM 25841 reduced diarrhea and enhanced growth performance by enhancing intestinal barrier function of weaned pigs experimentally infected with ETEC F18 (Kim et al., 2019; He et al., 2020). However, the impacts of this *Bacillus* strain on gut microbiota of weaned pigs under post-weaning diarrhea has yet to be addressed. Thus, the major objectives of the present study included: (1) to determine the impacts of ETEC infection on fecal and intestinal microbiome of weaned pigs; and (2) to investigate the effects of supplementing *B. subtilis* DSM 25841 on gut microbiome of ETEC infected pigs, in comparison to antibiotics, carbadox.

## Materials and methods

### Animals and study design

Animal procedures were reviewed and approved by the Institutional Animal Care and Use Committee (IACUC #19322) at the University of California, Davis (UC Davis). A total of 48 weaned pigs (21 day (d) old; 6.17 ± 0.36 kg) with equal number of barrows and gilts were obtained from the Swine Teaching and Research Center and the experiment was conducted at Cole facility at UC Davis. All pigs and their sows did not receive *E. coli* vaccines, antibiotic injections, or antibiotics in feed prior to the experiment. After weaning, pigs were individually housed (pen size: 0.61 m × 1.22 m) and assigned into one of 4 treatment groups using a randomized complete block design with sex normalized by body weight and litter as blocks and pig as experimental unit. Four treatments were: (1) negative control (NC): control diet and without *E. coli* challenge, (2) positive control (PC): control diet and with ETEC challenge, (3) AGP: inclusion of 50 mg/kg carbadox and with ETEC challenge, (4) DFM: inclusion of 500 mg/kg *B. subtilis* DSM 25841 (2.56 × 10^9^ CFU/kg) and with ETEC challenge. There were 12 replicate pigs per treatment. The experiment lasted 28 days with first 2 weeks as phase 1 and last 2 weeks as phase 2. Therefore, 6 diets were prepared and all diets met the current estimates for nutrient requirements for nursery pigs (NRC, 2012; Table 1).

**Table 1:** Ingredient compositions of experimental diets^1^.

Ingredient, %	Control, phase I	Control, phase II
Corn	44.41	57.27
Dried whey	15.00	10.00
Soybean meal	18.00	22.00
Fish meal	10.00	7.00
Lactose	6.00	–
Soy protein concentrate	3.00	–
Soybean oil	2.00	2.00
Limestone	0.56	0.70
L-Lysine·HCl	0.21	0.23
DL-Methionine	0.08	0.05
L-Threonine	0.04	0.05
Salt	0.40	0.40
Vit-mineral, Sow 6^2^	0.30	0.30
Total	100.00	100.00
Calculated energy and nutrient		
Metabolizable energy, kcal/kg	3,463	3,429
Net energy, kcal/kg	2,601	2,575
Crude protein, %	22.27	20.80
Arg,^3^ %	1.23	1.15
His,^3^ %	0.49	0.47
Ile,^3^ %	0.83	0.76
Leu,^3^ %	1.62	1.55
Lys,^3^ %	1.35	1.23
Met,^3^ %	0.45	0.39
Thr,^3^%	0.79	0.73
Trp,^3^ %	0.23	0.21
Val,^3^ %	0.91	0.84
Met + Cys,^3^ %	0.74	0.68
Phe + Tye,^3^ %	1.45	1.38
Ca, %	0.80	0.70
Total P, %	0.68	0.59
Digestible P, %	0.47	0.37
Analyzed nutrient, as-is		
Dry matter, %	90.70	89.90
Crude protein, %	23.13	21.30
ADF, %	7.26	9.35
NDF, %	2.54	3.60
Ca, %	0.96	0.88
P, %	0.71	0.59

^1^In each phase, two additional diets were formulated by adding probiotics or Carbadox to the control diet, respectively. The dose for probiotics was 500 mg/kg, which was equal to 2.56 × 10^9^ CFU *Bacillus subtilis*/kg diet. The dose for carbadox was 50 mg/kg diet.

^2^Provided the following quantities of vitamins and micro minerals per kilogram of complete diet: Vitamin A as retinyl acetate, 11,136 IU; vitamin D3 as cholecalciferol, 2,208 IU; vitamin E as DL-alpha tocopheryl acetate, 66 IU; vitamin K as menadione dimethylprimidinol bisulfite, 1.42 mg; thiamin as thiamine mononitrate, 0.24 mg; riboflavin, 6.59 mg; pyridoxine as pyridoxine hydrochloride, 0.24 mg; vitamin B12, 0.03 mg; D-pantothenic acid as D-calcium pantothenate, 23.5 mg; niacin, 44.1 mg; folic acid, 1.59 mg; biotin, 0.44 mg; Cu, 20 mg as copper sulfate and copper chloride; Fe, 126 mg as ferrous sulfate; I, 1.26 mg as ethylenediamine dihydriodide; Mn, 60.2 mg as manganese sulfate; Se, 0.3 mg as sodium selenite and selenium yeast; and Zn, 125.1 mg as zinc sulfate.

^3^Amino acids were indicated as standardized ileal digestible AA.

The experiment included a 7-day habituation period and 21 days after the first ETEC F18 inoculation (day 0). Pigs in the ETEC challenge groups received 3 oral doses of ETEC F18 at 10^10^ CFU per dose per day, while pigs in NC group were orally inoculated with 3 ml phosphate-buffered saline per day. The ETEC F18 were cultured in Dr. Xunde Li’s lab at Western institute for Food Safety & Security at UC Davis. The bacterial strain was originally isolated from a field disease outbreak by the University of Illinois Veterinary Diagnostic Lab (isolate number: U.IL-VDL # 05–27,242) and the strain expresses heat-labile toxin, heat-stable toxin b, and Shiga-like toxins. Our previous published research confirmed the current ETEC challenge dosage induced mild diarrhea in weaned pigs (Liu et al., 2013; Kim et al., 2019). The detailed animal study procedures and data for growth performance and diarrhea were reported in He et al. (2020).

### Sample collection

Prior to weaning, tail samples were collected from all piglets to assess their susceptibility to ETEC F18 using the genotyping analysis described in Kreuzer et al. (2013). All pigs used in the present study were susceptible to ETEC F18. Fresh fecal samples were collected from 7 pigs per treatment at the beginning of the experiment (day − 7), and from all pigs on day 0 before ETEC inoculation, and day 7 and day 21 post-inoculation (PI). Samples were immediately stored at −80°C until further analysis. At the termination of the experiment (day 21 PI), all pigs were euthanized. For euthanasia, pigs were anesthetized by intramuscularly injecting 1 ml mixture of telazol (100 mg) ketamine (50 mg), and xylazine (50 mg) prior to an intracardiac injection of 78 mg sodium pentobarbital (Vortech Pharmaceuticals, Ltd., Dearborn, MI) per 1 kg of body weight. Digesta was collected from the middle of jejunum (approximately equal length from pylorus to ileocecal junction), ileum (close to the ileocecal junction), and distal colon (prior to the rectum) and was immediately frozen into liquid nitrogen and stored at −80°C until further analysis. A total of 6 pigs were removed from the whole data set due to health issues after *E. coli* infection or as outliers, including 3 pigs from the PC group and 3 pigs from the AGP group (He et al., 2020).

### Library preparation

Bacterial DNA was extracted from 154 fecal samples and 126 intestinal digesta using the Quick-DNA Fecal/Soil Microbe Kit (Zymo Research, Irvine, CA, United States) according to the manufacturer’s instructions. DNA samples was amplified by PCR at the V4 region of the 16S rRNA gene using primers 515F (5′-XXXXXXXX**GT**GTGCCAGCMGCCGCGGTAA-3′), which included an 8-nt poly-X sequence indicating a barcode unique to each sample followed by an 2-nt Illumina adapter (bold), and 806R (5′-GGACTACHVGGGTWTCTAAT-3′) (Caporaso et al., 2012). Samples were prepared for PCR amplification in duplicates, and each PCR reaction was comprised of 2 μl template DNA, 9.5 μl nuclease free water, 12.5 μl GoTaq 2× Master Mix (Promega, Madison, WI, United States), 0.5 μl V4 reverse primer (10 μM), and 0.5 μl barcoded forward primer (10 μM). Amplification was performed in a thermocycler with the following setting: 94°C for 3 min for initializing denaturation; followed by 35 cycles of 94°C for 45 s, 50°C for 1 min, and 72°C for 1.5 min; and 72°C for 10 min for final elongation. Agarose gel electrophoresis was used to verify the amplicon size for each sample and band brightness was observed to subjectively quantify the amount of sample to be added when pooling DNA amplication products. Pooled sample was then purified using the QIAquick PCR Purification Kit (Qiagen, Hilden, Germany) and submitted to the UC Davis Genome Center DNA Technologies Core for 250 bp paired-end sequencing on the Illumina MiSeq platform (Illumina, Inc., San Diego, CA, United States).

### Microbiota analysis

Raw fastq files were first demultiplexed and barcode sequences were removed using the software saber^1^ and demultiplexed sequences were then imported into Quantitative Insights into Microbial Ecology 2 (QIIME2; version 2019.4; Bolyen et al., 2019, 2). Using the DADA2 plugin, primers and lower quality reads were removed and the paired end reads were denoised and merged (Callahan et al., 2016, p. 2). Chimeras were removed after merging and amplicon sequence variants (ASVs) were constructed as well. Representative sequences for each ASV were aligned using MAFFT and masked alignments were used to generate phylogenetic trees using FastTree2 (Price et al., 2010; Katoh and Standley, 2013). Python library scikit-learn was used to assign taxonomy based on representative sequences against Silva (version 138), which was pre-trained in QIIME2 and clipped to only the V4 hypervariable region and clustered at 99% sequence identity (Pedregosa et al., 2011; Quast et al., 2012; Bokulich et al., 2018).

Shannon and Chao1 indices were measured for alpha diversity by using the estimate_richness function in phyloseq (McMurdie and Holmes, 2013). To compare community composition among treatments and day or intestinal site, Bray-Curtis matrix was measured to calculate beta diversity. Relative abundance of each taxon in each sample was calculated by dividing the taxa count by total number of filtered reads within each sample.

### Statistical analyzes

Files were exported from QIIME2 and imported into the R 4.1.0 for data visualization and statistical analysis (Team, 2021). All microbiota analyzes was performed using the phyloseq package and data were visualized using the ggplot2 package (Wickham, 2011). Normality and homoscedasticity were tested using the Shapiro Wilks test and Bartlett test, respectively. Linear mixed-effect model was fitted using the lme4 package with treatment and site or day and interaction as fixed effects while pig as random effect (Bates et al., 2014, p. 4). Significance of each term in the model was determined using the F-test as type 3 analysis of variance using the Anova function in the car package, followed by group comparison using the cld function in the emmeans package (Fox and Weisberg, 2018; Lenth, 2021). When normality or homoscedasticity was not observed, non-parametric test was performed using the Kruskal-Wallis sum-rank test using the agricolae package (de Mendiburu and de Mendiburu, 2019). Bray–Curtis dissimilarity was first tested for homoskedasticity using the betadisper function and the statistical significance was tested using PERMANOVA and the vegan package (Oksanen et al., 2013). Statistical significance was assessed at α = 0.05 and statistical tendency at *α* = 0.10, and *p-*values were adjusted for multiple comparisons using false discovery rate (FDR).

## Results

### Shifts in fecal microbiota with age

**Figure 1:**
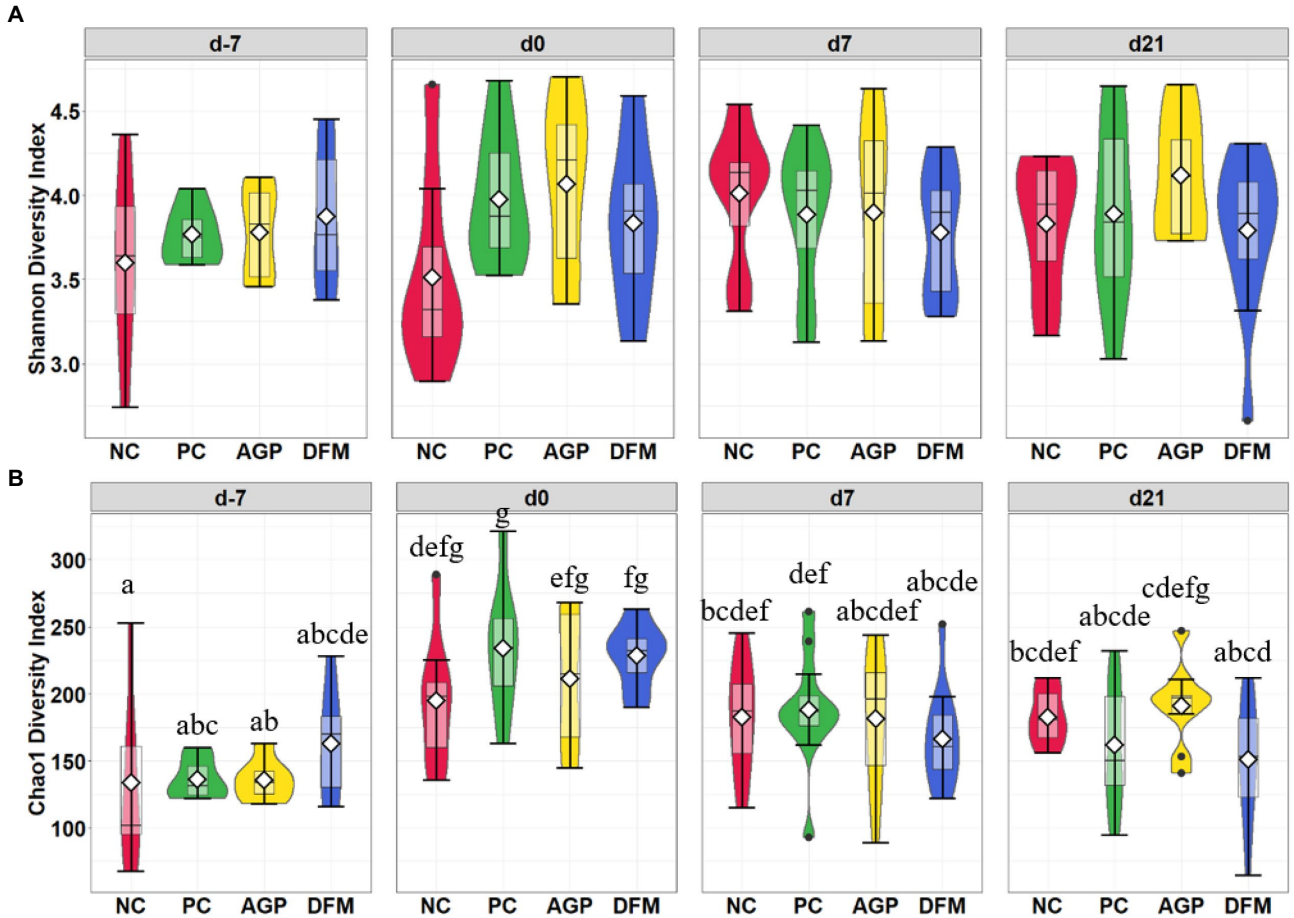
Alpha diversity as indicated by Shannon **(A)** and Chao1 **(B)** indices in feces collected from enterotoxigenic *Escherichia coli* challenged pigs fed diets supplemented with antibiotics (AGP) or *Bacillus subtilis* (DFM) at the beginning of the experiment, on day 0 before inoculation (day − 7), on day 7 and 21 post-inoculation. No difference was observed in Shannon **(A)**. NC = negative control, PC = positive control, AGP = antibiotics, DFM = *B. subtilis*. Violin plots are colored by diet. Data are expressed as mean (diamond) ± SEM. ^a-g^Means without a common superscript are different (*p* < 0.05). Each mean represents 7 observations on day −7 and each mean represents 9–12 observations on day 0 and day 7 and 21 post-inoculation.

**Figure 2:**
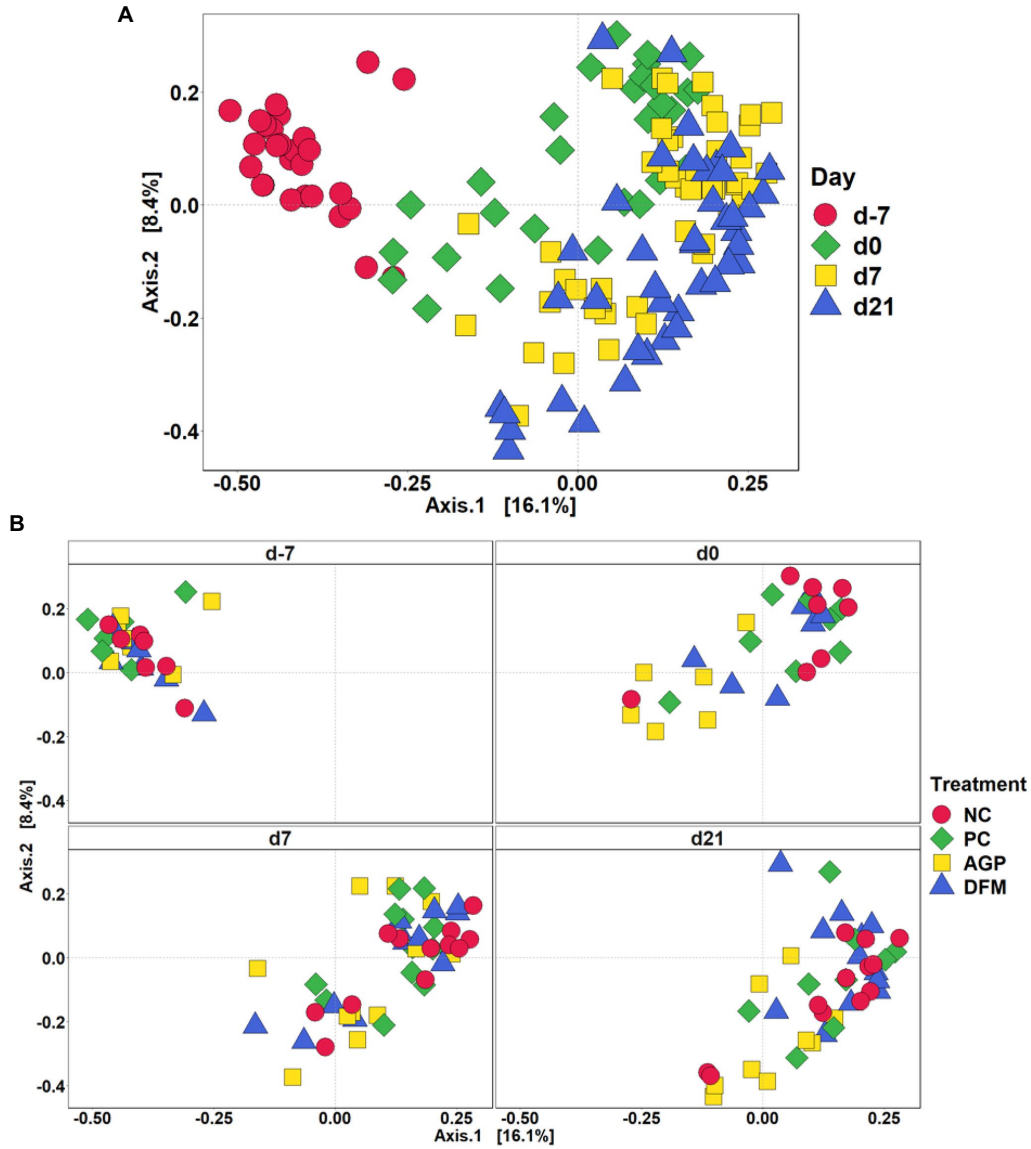
Beta diversity of fecal microbiota in enterotoxigenic *E. coli* F18 challenged pigs at the beginning of the experiment, on day 0 before inoculation, on day 7 and 21 post-inoculation by day **(A)** and treatment **(B)**. Data were analyzed by principal coordinate analysis (PCoA) based on the Bray–Curtis dissimilarity. NC = negative control, PC = positive control, AGP = antibiotics, DFM = *B. subtilis*. Each mean represents 7 observations on day −7 and each mean represents 9–12 observations on day 0 and day 7 and 21 post-inoculation.

After quality filtering in QIIME2, a total of 3,908,518 filtered sequences were obtained from 273 samples. The median reads per sample was 14,338 and the total number of taxa discovered was 3,430. No significant difference in Shannon index was observed among treatments throughout the experiment (Figure 1A). Sampling days influenced (*p* < 0.05) Chao1 index and an increase (*p* < 0.05) in Chao1 index was observed in all treatments on day 0 than day − 7 (Figure 1B). Feces collected from pigs in the PC and DFM groups had decreased (*p* < 0.05) Chao1 index on day 7 PI, compared with feces collected on day 0. The principal coordinate analysis (PCoA) plot visualized dissimilarities between samples employing Bray-Curtis distance matrix. The samples grouped according to sampling days, as indicated by the statistical difference using adonis2 (*R*^2^ = 0.20; *p* < 0.05) (Figure 2A). No distinctive clusters were observed among treatments on day − 7. However, a separated cluster among treatments was observed on day 0 (*R*^2^ = 0.15; *p* < 0.05), day 7 PI (*R*^2^ = 0.10; *p* < 0.05) and day 21 PI (*R*^2^ = 0.14; *p* < 0.05; Figure 2B).

Relative abundances of various phyla and families are presented in Table 2. Firmicutes and Bacteroidetes were the two most abundant phyla in feces throughout the experiment, accounting for more than 75% in relative abundance per treatment group on each sampling day. No difference was observed in the relative abundance of top 6 phyla in feces among all treatments on day − 7. The relative abundance of Firmicutes was increased (*p* < 0.05), while the relative abundance of Bacteroidetes was decreased (*p* < 0.05) in NC as the pig age was increased. No difference was observed in the relative abundance of Spirochaetes, Actinobacteria, and Euryarchaeota among treatments and sampling days. On day 0 and day 7 PI, pigs in NC and DFM had greater (*p* < 0.05) relative abundance of Firmicutes in feces than pigs in PC and AGP. At the concluding day of the experiment (day 21 PI), pigs in the AGP group had lower (77.22% vs. 82.36%, *p* < 0.05) relative abundance of Firmicutes in feces than pigs in NC. No difference was observed in the relative abundance of Bacteroidetes between NC and PC on any sampling date. The relative abundance of Bacteroidetes was lower (10.03% vs. 18.31%, *p* < 0.05) in feces of pigs fed with DFM than pigs supplemented with AGP on day 7 PI. Pigs in PC had greater (*p* < 0.05) relative abundance of Proteobacteria in feces than pigs in NC on day 7 PI. No difference was observed in the relative abundance of Proteobacteria between AGP and DFM throughout the experiment.

**Table 2:** Relative abundance (%) of Firmicutes, Bacteroidetes, and Proteobacteria and their top families in feces of enterotoxigenic *Escherichia coli* challenged pigs fed diets supplemented with antibiotics (AGP) or *Bacillus subtilis* (DFM).

	Day 7				Day 0				Day 7 post-inoculation				Day 21 post-inoculation			
	Negative control	Positive control	AGP	DFM	Negative control	Positive control	AGP	DFM	Negative control	Positive control	AGP	DFM	Negative control	Positive control	AGP	DFM
Firmicutes	55.00^g^	54.71^g^	61.76^fg^	56.61^g^	77.84^abcd^	72.20^bcdef^	64.34^fg^	75.93^abcde^	79.17^abc^	71.11^def^	65.40^efg^	80.11^ab^	82.36^a^	76.09^abcd^	71.99^cdef^	77.22^abcd^
*Bacillaceae*	0.00^bc^	0.00^bc^	0.00^bc^	0.00^bc^	0.00^bc^	0.00^bc^	0.00^bc^	0.03^a^	0.00^bc^	0.00^bc^	0.00^bc^	0.01^c^	0.00^bc^	0.00^bc^	0.00^c^	0.05^ab^
*Christensenellaceae*	0.38^b^	1.53^ab^	2.15^ab^	4.18^a^	1.27^ab^	1.73^ab^	3.09^ab^	1.72^ab^	1.23^ab^	0.90^ab^	1.14^ab^	2.28^ab^	0.71^b^	0.95^ab^	0.76^ab^	0.48^b^
*Clostridiaceae1*	2.53^abc^	3.68^ab^	4.01^ab^	6.74^a^	0.24^c^	0.49^bc^	2.73^abc^	1.04^abc^	1.69^bc^	1.14^bc^	4.29^ab^	3.55^abc^	6.73^ab^	1.93^abc^	12.47^ab^	2.22^bc^
*Lachnospiraceae*	16.59^bcde^	10.26^de^	11.94^cde^	6.58^e^	17.98^bcd^	26.26^ab^	26.87^ab^	23.64^ab^	27.21^a^	23.07^ab^	20.04^abcd^	23.88^ab^	21.83^ab^	21.32^abc^	18.69^bcd^	20.06^abc^
*Lactobacillaceae*	11.55^cde^	16.92^bcde^	21.54^bcd^	15.93^bcde^	39.56^a^	24.55^abc^	6.42^de^	27.09^abc^	23.10^abc^	28.21^ab^	20.73^bc^	27.91^ab^	14.63^cde^	14.24^cde^	4.44^e^	23.98^bc^
*Peptostreptococcaceae*	1.46^abcd^	2.29^ab^	0.93^abcd^	1.92^ab^	0.16^e^	0.12^e^	0.14^e^	0.38^de^	0.65^bcd^	0.53^cde^	3.33^abc^	1.69^abc^	3.81^a^	1.10^abcd^	6.91^a^	1.21^bcd^
*Ruminococcaceae*	14.27	12.96	12.58	10.48	11.23	12.55	17.97	13.88	18.63	10.89	11.30	12.94	15.07	16.62	15.29	14.57
*Streptococcaceae*	0.64^bc^	1.11^ab^	0.39^bcd^	0.61^bcd^	0.05^d^	0.08^cd^	0.02^d^	0.2^cd^	0.18^cd^	1.05^bcd^	0.38^bcd^	2.97^bcd^	10.44^a^	6.27^ab^	0.10^cd^	1.90^b^
*Veillonellaceae*	2.32^def^	0.67^f^	1.22^ef^	1.21^ef^	4.08^cdef^	3.41^bcd^	2.64^def^	3.46^bcde^	3.77^bcd^	2.38^def^	1.87^def^	2.56^def^	6.69^abc^	10.93^a^	9.11^bcd^	9.99^ab^
Bacteroidetes	29.43^ab^	31.97^a^	21.97^abc^	20.62^abcd^	11.12^cde^	14.20^cde^	17.96^abcd^	16.04^bcde^	12.27^cde^	12.65^cde^	18.31^abc^	10.03^e^	9.70^e^	10.25^de^	15.85^bcde^	13.73^cde^
*Bacteroidaceae*	18.07^a^	15.14^ab^	8.57^ab^	6.87^abc^	0.65^cde^	0.51^ef^	1.00^bcd^	0.68^cde^	0.11^fgh^	0.85^de^	2.2^cd^	0.28^efg^	0.01^h^	0.22^gh^	0.16^efg^	0.01^h^
*Muribaculaceae*	0.50	1.83	0.79	0.79	2.13	3.75	4.20	3.63	2.50	1.83	2.36	1.24	2.40	1.64	3.05	2.52
*Prevotellaceae*	6.19	5.87	4.91	5.34	6.38	6.99	8.07	8.32	7.87	8.02	11.54	7.36	6.54	7.62	10.00	10.15
*Rikenellaceae*	3.51^ab^	5.88^a^	5.36^ab^	5.39^ab^	1.12^bcde^	2.08^abc^	3.92^ab^	2.80^abc^	1.31^bcde^	1.49^bcde^	1.63^bcde^	0.87^de^	0.61^e^	0.65^e^	2.27^abcd^	0.97^cde^
*Tannerellaceae*	0.41^ab^	1.88^a^	1.36^a^	1.17^ab^	0.32^ab^	0.31^bcd^	0.26^bcd^	0.47^ab^	0.13^cde^	0.03^e^	0.12^bcde^	0.11^bcd^	0.04^de^	0.05^de^	0.22^bc^	0.03^de^
Proteobacteria	8.21^abc^	6.72^ab^	3.78^abc^	5.92^ab^	1.52^abc^	1.86^abc^	2.82^abc^	2.72^abc^	1.18^c^	7.38^a^	8.88^abc^	2.45^abc^	2.25^bc^	6.60^ab^	5.26^abc^	2.46^bc^
*Desulfovibrionaceae*	0.60^ab^	1.14^a^	1.46^a^	1.09^ab^	0.34^abcd^	0.32^bcde^	0.33^bcde^	0.38^abc^	0.17^def^	0.30^bcde^	0.24^cdef^	0.22^def^	0.16^ef^	0.14^ef^	0.16^def^	0.13^f^
*Enterobacteriaceae*	6.07^bcd^	4.23^abc^	1.97^abc^	3.60^ab^	0.49^de^	0.79^cd^	0.60^bcd^	1.75^abc^	0.69^cd^	6.49^a^	8.35^ab^	1.94^abc^	0.10^de^	0.03^e^	0.01^e^	0.01^e^
*Pasteurellaceae*	0.22^ab^	1.27^abcd^	0.09^abc^	0.19^a^	0^d^	0^d^	0^cd^	0^cd^	0^cd^	0.01^bcd^	0^d^	0^cd^	0^cd^	0.01^bcd^	0^d^	0^d^
*Succinivibrionaceae*	0.58^ef^	0^f^	0.01^f^	0.87^ef^	0.65^cde^	0.52^bcd^	1.76^cde^	0.54^bcde^	0.28^def^	0.50^cde^	0.17^def^	0.20^def^	1.56^cde^	6.12^a^	5.07^ab^	2.30^abc^

^a–g^Means without a common superscript are different (*p* < 0.05). Each mean represents 7 observations on day −7 and each mean represents 9–12 observations on day 0 and day 7 and 21 post-inoculation.

Within Firmicutes phylum, the relative abundance of *Bacillaceae* was the greatest (*p* < 0.05) in DFM among all treatment on day 0, 7, and 21 PI. The relative abundance of *Lactobacillaceae* in NC was increased (*p* < 0.05) in feces on day 0 but decreased (*p* < 0.05) on day 21 PI, compared with feces collected on day − 7 (Table 2). In addition, pigs supplemented with AGP had reduced (*p* < 0.05) relative abundance of *Lactobacillaceae* in feces on day 0 and day 21 PI. The relative abundance of *Lactobacillaceae* was lower (*p* < 0.05) in feces of pigs fed with AGP than that in DFM (4.44% vs. 23.98%) on day 21 PI. Pigs in PC, AGP, and DFM had increased (*p* < 0.05) relative abundance of *Lachnospiraceae* from day − 7 to 0. *Bacteroidaceae* was the most abundant family in Bacteroidetes phylum on day − 7, while *Prevotellaceae* was the most abundant one on day 0, 7, and day 21 PI (Table 2). Pigs in AGP had greater (*p* < 0.05) relative abundance of *Bacteroidaceae* in feces than pigs in NC and DFM on day 7 and day 21 PI. Within Proteobacteria phylum, the relative abundance of *Enterobacteriaceae* was decreased (*p* < 0.05) but the relative abundance of *Succinivibrionaceae* was increased (*p* < 0.05), as pig age was increased (Table 2). Pigs in PC and AGP had greater (*p* < 0.05) relative abundance of *Enterobacteriaceae* than NC on day 7 PI. However, no difference was observed in the relative abundance of *Enterobacteriaceae* among other treatments on day 7 PI and other sampling dates. The relative abundance of *Succinivibrionaceae* was higher (*p* < 0.05) in PC and AGP than pigs in NC on day 21 PI.

**Figure 3:**
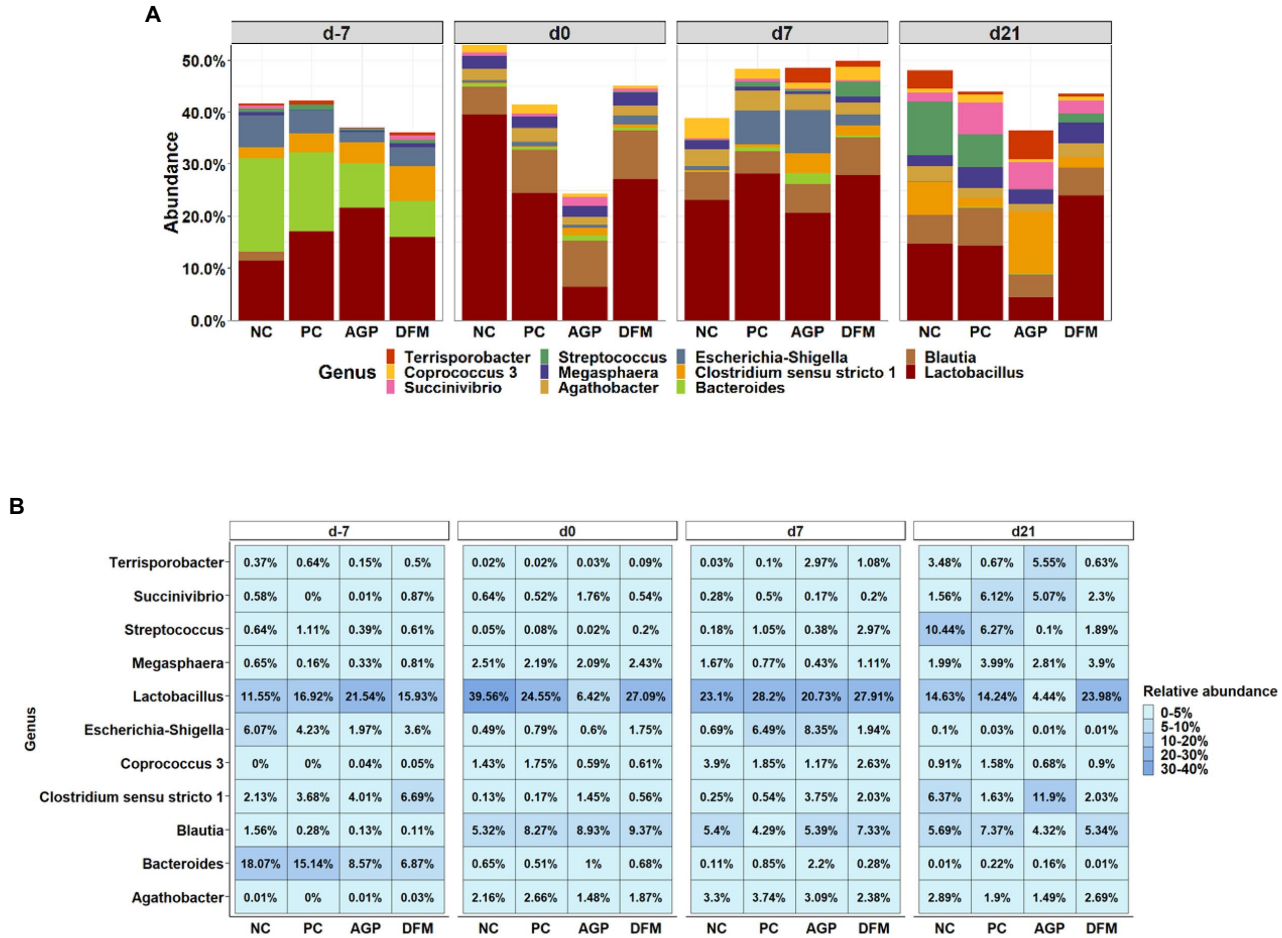
Relative abundance of genus that were most abundant in fecal microbiota of piglets visualized in bar plot **(A)** and heatmap **(B)**. Each mean represents 7 observations on day −7 and each mean represents 9–12 observations on day 0 and day 7 and 21 post-inoculation.

The top 11 abundant genera in feces are presented in Figure 3. From day − 7 to day 21 PI, all treatments had increased (*p* < 0.05) relative abundance of *Blautia* and reduced (*p* < 0.05) relative abundance of *Bacteroides* and *Escherichia-Shigella*. All treatments, except AGP, had reduced (*p* < 0.05) relative abundance of *Megasphaera* from day −7 to day 21 PI. The most abundant genus was *Lactobacillus*. In NC pigs, the relative abundance of *Lactobacillus* was increased (*p* < 0.05) from day −7 to 0, and then decreased (*p* < 0.05) by day 21 PI. Pigs in AGP had the lowest abundance (*p* < 0.05) of *Lactobacillus* on day 0 and pigs in DFM had the greatest abundance (*p* < 0.05) of *Lactobacillus* on day 21 PI among treatments. In addition, the relative abundance of *Lactobacillus* was greater in DFM (27.91% vs. 20.73%, *p* < 0.05) than in AGP on day 7 PI. The relative abundance of *Blautia* was greater (*p* < 0.05) in feces collected on day 0, 7 PI, and day 21 PI than in feces collected on day −7. The relative abundance of *Bacteroides* was greater (*p* < 0.05) in AGP than in PC on day 0 and was greater (*p* < 0.05) in AGP than in DFM on day 7 and day 21 PI.

### Shifts in gut microbiota within different intestinal sites

**Figure 4:**
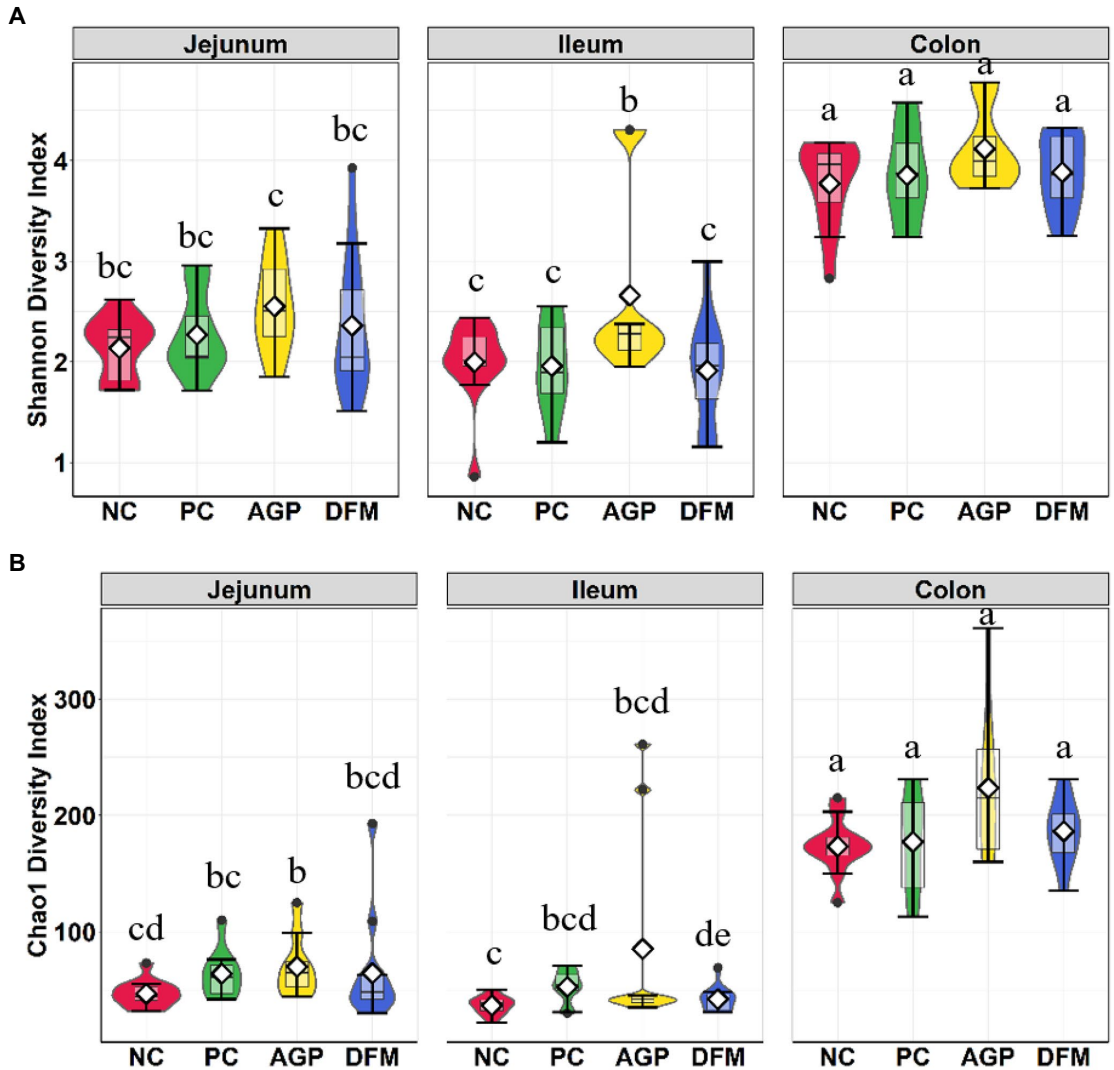
Alpha diversity as indicated by Shannon **(A)** and Chao1 **(B)** indices in intestinal digesta collected from weaned pigs challenged with enterotoxigenic *E. coli*. Pigs were supplemented with antibiotics (AGP) or *B. subtilis* (DFM). NC = negative control, PC = positive control. Violin plots are colored by diet. Data are expressed as mean (diamond) ± SEM. ^a-g^Means without a common superscript are different (*p*  <  0.05). Each mean represents 9–12 observations.

The microbial composition in the digesta of jejunum, ileum, and colon was also investigated at the conclusion of this experiment (day 21 PI). Significant interaction between treatment and intestinal site was observed (*p* < 0.001) in both Shannon and Chao1 diversities (Figure 4). Shannon and Chao1 indices in colon digesta were greater (*p* < 0.05) than in jejunal and ileal digesta. No difference was observed in Shannon and Chao1 indices between NC and PC in any of intestinal sites. Pigs supplemented with AGP had greater (*p* < 0.05) Shannon index than other treatments in the ileum (Figure 4A) and had greater (*p* < 0.05) Chao1 index in the jejunum than NC (Figure 4B). Supplementation of DFM reduced (*p* < 0.05) Chao 1 index in ileal digesta when compared with NC.

**Figure 5:**
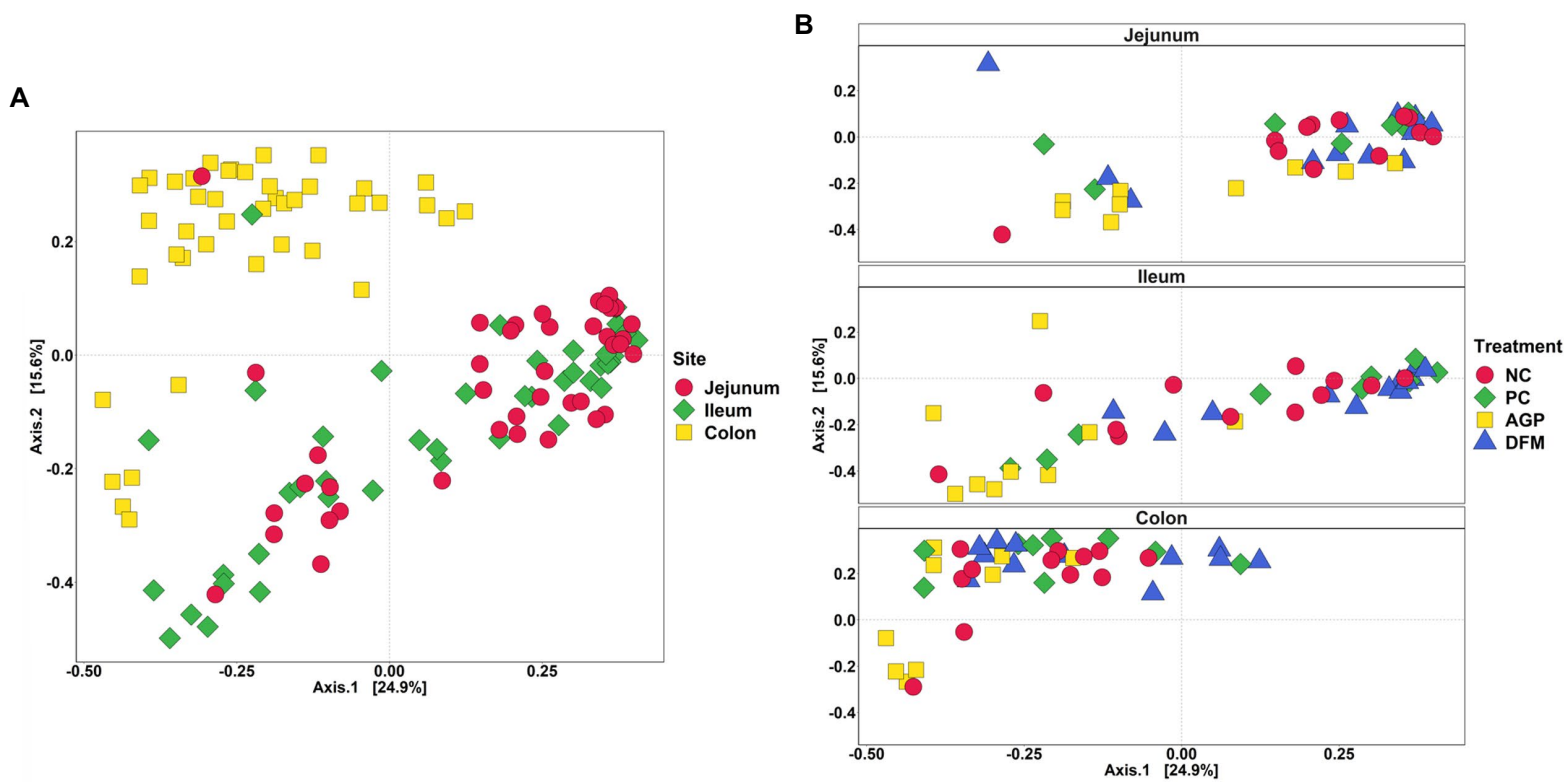
Beta diversity of intestinal digesta microbiota in pigs challenged with enterotoxigenic *E. coli* by intestinal site **(A)** and treatment **(B)**. Data were analyzed by principal coordinate analysis (PCoA) based on Bray–Curtis dissimilarity. NC = negative control, PC = positive control, AGP = antibiotics, DFM = *B. subtilis*. Each mean represents 9–12 observations.

The adonis2 test demonstrated significance in treatment (*R*^2^ = 0.10, *p* < 0.05), intestinal site (*R*^2^ = 0.18, *p* < 0.05), and treatment and intestinal site interaction (*R*^2^ = 0.05, *p* < 0.05). In Figure 5A, all colon samples were clustered together and were separated from jejunal and ileal samples, whereas clusters for ileum and jejunum were indistinguishable from each other. No clear separation was observed among treatments in jejunum, while the AGP cluster was distinct from DFM cluster in ileum (Figure 5B). Within colon samples, AGP cluster partially overlapped with DFM cluster, while NC and PC clusters were overlapping with each other.

The relative abundance of phyla, families, and genera are presented in Table 3. Firmicutes, Bacteroidetes, Actinobacteria, and Proteobacteria were the top four abundant phyla in the intestinal tract of weaned pigs. Firmicutes was the most abundant phylum in jejunum, ileum, and colon in all pigs. Unlike fecal samples, Bacteroidetes was second most abundant phylum in colon, Actinobacteria was the second most abundant phylum in jejunum, and Proteobacteria was the second most abundant phylum in ileum. No difference was observed in the relative abundance of phyla between NC and PC in all intestinal sites. The relative abundance of Bacteroidetes in ileal digesta was greater (*p* < 0.05) in AGP than DFM. The relative abundance of Actinobacteria in ileal digesta was greater (*p* < 0.05) in PC than AGP. Pigs in AGP had the greatest (*p* < 0.05) relative abundance of Proteobacteria in jejunal digesta among all treatments and had greater (*p* < 0.05) relative abundance of Proteobacteria in ileal digesta than pigs in DFM (4.12% vs. 0.22%).

**Table 3:** Relative abundance (%) of Firmicutes, Bacteroidetes, Actinobacteria, and Proteobacteria and their top families in intestinal digesta of enterotoxigenic *E. coli* challenged pigs fed diets supplemented with antibiotics (AGP) or *B. subtilis* (DFM).

	Jejunal digesta				Ileal digesta				Colon digesta			
	Negative control	Positive control	AGP	DFM	Negative control	Positive control	AGP	DFM	Negative control	Positive control	AGP	DFM
Firmicutes	93.20^ab^	84.11^bcde^	89.51^abc^	91.85^abc^	93.28^a^	92.77^abc^	86.08^abcd^	95.67^a^	85.64^cde^	80.75^de^	74.89^e^	81.23^de^
*Bacillaceae*	0.03^cde^	0.07^cd^	0.27^b^	0.64^a^	0.00^e^	0.06^cde^	0.02^cde^	0.18^ab^	0.00^e^	0.00^e^	0.00^de^	0.02^c^
*Clostridiaceae1*	2.89^bc^	1.78^c^	4.91^bc^	3.59^bc^	11.89^a^	11.96^ab^	22.22^a^	2.88^abc^	7.22^ab^	1.46^bc^	13.56^ab^	2.35^bc^
*Lachnospiraceae*	0.46^bc^	1.52^b^	0.43^bc^	2.55^bc^	0.13^d^	0.42^bc^	2.04^cd^	0.09^d^	23.19^a^	23.15^a^	21.93^a^	24.36^a^
*Lactobacillaceae*	76.09^a^	60.26^ab^	59.44^ab^	70.54^a^	47.48^b^	56.22^ab^	22.98^c^	77.12^a^	15.09^cd^	14.42^cd^	4.11^d^	21.20^c^
*Peptostreptococcaceae*	3.82^bc^	0.06^c^	3.50^ab^	2.14^bc^	9.59^a^	5.24^ab^	15.18^a^	3.82^ab^	3.96^a^	0.86^ab^	7.48^a^	1.42^ab^
*Ruminococcaceae*	0.06^cde^	0.44^bc^	0.13^b^	1.55^cde^	0.01^de^	0.04^cde^	2.59^bcd^	0.01^e^	15.52^a^	18.15^a^	15.24^a^	15.75^a^
*Streptococcaceae*	4.93^abc^	9.61^ab^	18.40^a^	5.69^abc^	19.42^a^	12.98^abc^	11.01^abc^	8.94^abc^	10.78^a^	6.60^bcd^	0.09^d^	3.21^cd^
*Veillonellaceae*	4.40^cd^	6.30^abcd^	0.09^g^	3.85^cde^	1.38^efg^	1.98^def^	1.21^fg^	0.93^fg^	6.63^abc^	12.32^a^	8.09^bcd^	9.81^ab^
												
Bacteroidetes	0.03^bc^	0.02^bc^	0.01^bc^	1.08^bc^	0^bc^	0.03^bc^	4.16^b^	0^c^	7.25^a^	7.35^a^	12.41^a^	9.96^a^
*Bacteroidaceae*	0^b^	0^b^	0^b^	0^b^	0^b^	0^b^	0.06^b^	0^b^	0.01^b^	0.11^b^	0.16^a^	0.01^b^
*Muribaculaceae*	0^c^	0.01^c^	0.01^c^	0.11^bc^	0^c^	0^c^	0.49^b^	0^c^	2.22^a^	1.74^a^	1.94^a^	2.09^a^
*Prevotellaceae*	0.03^bc^	0.01^b^	0^c^	0.94^bc^	0^c^	0.03^bc^	2.98^b^	0^bc^	4.64^a^	5.09^a^	8.93^a^	7.23^a^
*Rikenellaceae*	0^d^	0^cd^	0^d^	0.03^cd^	0^d^	0.01^cd^	0.54^c^	0^d^	0.28^ab^	0.35^b^	1.17^a^	0.57^ab^
												
Actinobacteria	4.76^a^	9.43^a^	1.79^abc^	4.37^a^	2.93^abc^	4.38^ab^	0.36^c^	3.35^abc^	2.26^abc^	2.99^abc^	0.5^bc^	2.48^ab^
*Actinomycetaceae*	0.01^bc^	0.14^a^	0.07^b^	0.05^b^	0^c^	0.06^b^	0^c^	0.01^bc^	0^c^	0^c^	0^c^	0^c^
*Atopobiaceae*	0.43^ab^	0.54^a^	0^b^	0.32^ab^	0.13^ab^	0.17^ab^	0.07^ab^	0.09^ab^	0.36^ab^	1.45^ab^	0.28^ab^	1.01^ab^
*Bifidobacteriaceae*	4.22^a^	8.21^a^	0.04^b^	3.81^a^	2.76^a^	3.94^a^	0.11^b^	3.20^a^	1.67^a^	1.10^ab^	0.01^b^	1.22^a^
*Coriobacteriaceae*	0^c^	0^c^	0^c^	0.02^c^	0^c^	0^c^	0.05^c^	0^c^	0.21^ab^	0.38^a^	0.20^b^	0.22^ab^
*Eggerthellaceae*	0.06^ab^	0.21^a^	0^b^	0.05^ab^	0.03^ab^	0.05^ab^	0^b^	0.02^ab^	0.02^ab^	0.05^ab^	0.01^ab^	0.03^ab^
*Micrococcaceae*	0.04^c^	0.32^ab^	1.51^a^	0.11^bc^	0.01^d^	0.15^bc^	0.12^bc^	0.03^c^	0^d^	0^d^	0^d^	0^d^
												
Proteobacteria	1.89^b^	0.4^b^	4.12^a^	0.22^b^	3.77^ab^	2.21^ab^	7.61^a^	0.92^b^	1.69^ab^	4.84^a^	4.6^a^	2.08^a^
*Burkholderiaceae*	0^a^	0^a^	0.04^a^	0^a^	0^a^	0^a^	0.41^a^	0^a^	0^a^	0.01^a^	0^a^	0^a^
*Desulfovibrionaceae*	0^b^	0^b^	0^b^	0.01^b^	0^b^	0^b^	0.04^b^	0^b^	0.07^a^	0.1^a^	0.14^a^	0.11^a^
*Enterobacteriaceae*	0.17^c^	0.02^abc^	0.04^abc^	0.01^c^	3.56^a^	0.64^ab^	0.71^bc^	0.04^bc^	0.07^abc^	0.01^c^	0.01^c^	0.01^c^
*Pasteurellaceae*	1.7^bc^	0.29^bcd^	3.75^a^	0.02^bcd^	0.21^bcd^	1.54^ab^	4.85^a^	0.87^bc^	0^cd^	0.01^bcd^	0^cd^	0^d^
*Succinivibrionaceae*	0^d^	0.01^cd^	0.09^bc^	0.15^cd^	0^d^	0^cd^	1.5^bc^	0^d^	1.41^b^	4.71^a^	4.45^a^	1.94^a^

^a–g^Means without a common superscript are different (*p* < 0.05). Each mean represents 7 observations on day −7 and each mean represents 9–12 observations on day 0 and day 7 and 21 post-inoculation.

Under family level, jejunal digesta contained highest (*p* < 0.05) relative abundance of *Lactobacillaceae*, *Actinomycetaceae*, and *Micrococcaceae* among three intestinal sites. Ileal digesta had more (*p* < 0.05) relative abundance of *Clostridiaceae1* and *Enterobacteriaceae* than jejunal and colon digesta. Colon digesta contained more (*p* < 0.05) *Lachnospiraceae*, *Ruminococcaceae*, *Veillonellaceae*, *Coriobacteriaceae*, *Succinivibrionaceae*, and top 4 families under Bacteroidetes than jejunal and ileal digesta. The relative abundance of *Lactobacillaceae* was greater (*p* < 0.05) in DFM than NC and AGP in ileal digesta and was greater (*p* < 0.05) in DFM than AGP in colon digesta. ETEC F18 challenge increased (*p* < 0.05) the relative abundance of *Actinomycetaceae* and *Micrococcaceae* in jejunal and ileal digesta, *Lachnospiraceae* in ileal digesta and *Succinivibrionaceae* in colon digesta, but reduced (*p* < 0.05) *Streptococcaceae* in colon digesta. Compared with PC, supplementation of AGP enhanced (*p* < 0.05) the relative abundance of *Peptostreptococcaceae* and *Pasteurellaceae* in jejunal digesta, *Muribacelaceae* in ileal digesta, and *Bacteroidaceae* and *Rikenellacea*e in colon digesta. In addition, inclusion of AGP reduced (*p* < 0.05) the relative abundance of *Veillonellaceae*, *Actinomycetaceae*, *Atopobiaceae*, *Bifidobacteriaceae*, *Eggerthellaceae* in jejunal digesta, *Lachnospiraceae*, *Actinomycetaceae*, *Bifidobacteriaceae* in ileal digesta, and *Veillonellaceae* and *Coriobacteriaceae* in colon digesta. In comparison to PC, supplementation of DFM reduced (*p* < 0.05) the relative abundance of *Actinomycetaceae* in jejunal digesta and *Lachnospiraceae* in ileal digesta. Pigs fed with DFM had greater (*p* < 0.05) the relative abundance of *Ruminococcaceae*, *Veillonellaceae*, *Bifidobacteriaceae* in jejunal digesta, *Lactobacillaceae* in both ileum and colon, and *Bifidobacteriaceae* in colon when compared with pigs in AGP. However, pigs fed with DFM had reduced (*p* < 0.05) the relative abundance of *Pasteurellaceae* in jejunum, *Ruminococcaceae*, *Rikenellaceae*, *Pasteurellaceae*, *Succinivibrionaceae* in ileum, and *Bacteroidaceae* in colon, compared with pigs fed with AGP. In all intestinal sites, pigs in DFM had the highest (*p* < 0.05) relative abundance of *Bacillaceae* among all treatments.

**Figure 6:**
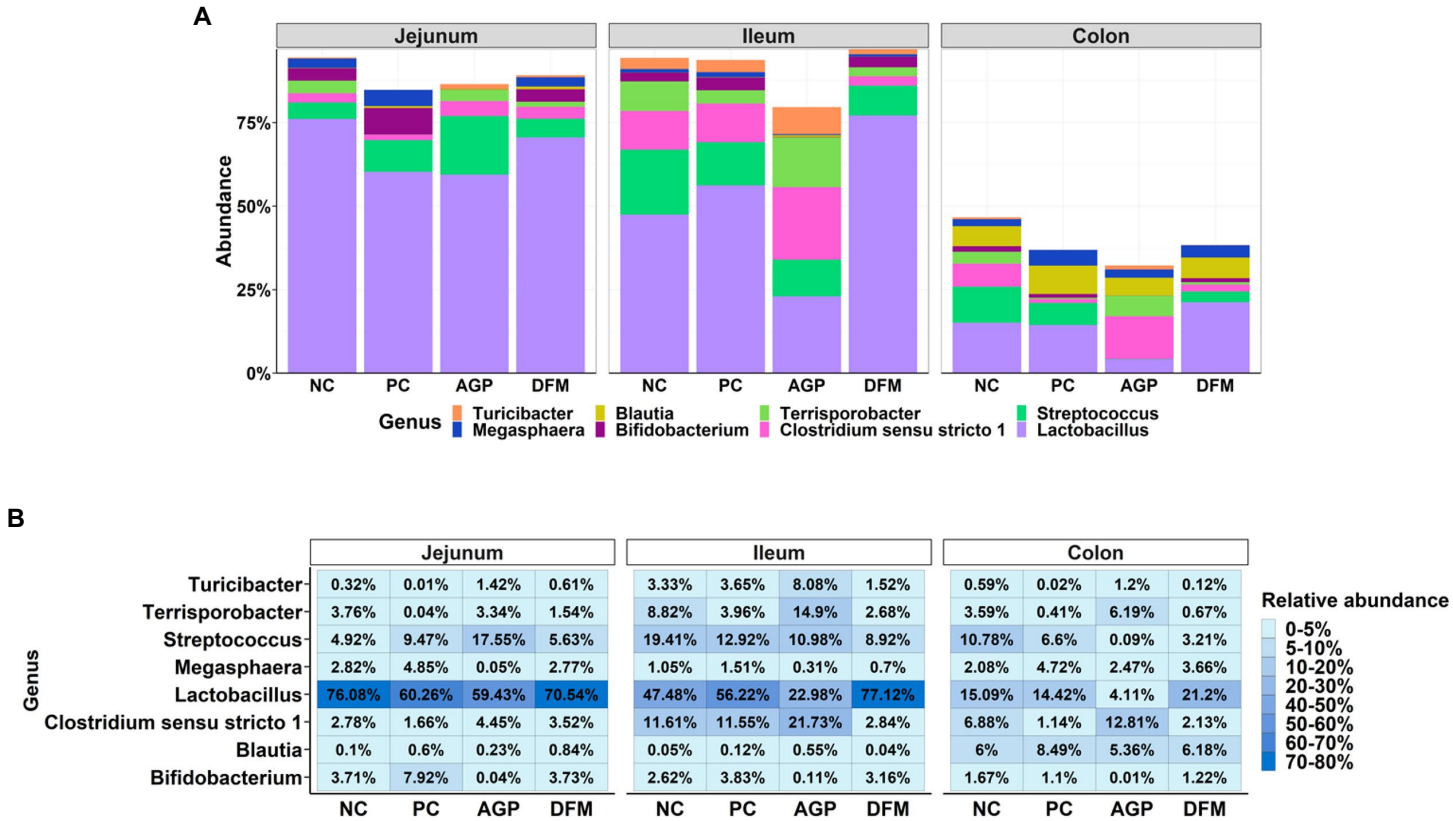
Relative abundance of top 8 genus in intestinal microbiota of piglets visualized in bar plot **(A)** and heatmap **(B)**. Each mean represents 9–12 observations.

The relative abundances of top abundant genera are presented in Figure 6. Relative abundance of *Lactobacillus* was greater (*p* < 0.05) but the relative abundance of *Clostridium sensu stricto 1* was less (*p* < 0.05) in ileal and colon digesta of pigs fed with DFM than in AGP. ETEC infection reduced the relative abundance of *Streptococcus* in ileum and colon, however, pigs supplemented with AGP had more (*p* < 0.05) *Streptococcus* in jejunum but lower (*p* < 0.05) *Streptococcus* in ileum and colon when compared with NC.

## Discussion

Our previous studies reported that dietary supplementation of *B. subtilis* DSM 25841 promoted growth performance, reduced diarrhea, and enhanced intestinal immunity in weaned piglets under ETEC challenge (Kim et al., 2019; He et al., 2020). However, the potential mechanisms of the beneficial effects of *B. subtilis* on swine health and the impacts of this *B. subtilis* strain on intestinal microbiota remain unclear. In the present study, we characterized the impacts of ETEC F18 infection, *B. subtilis* DSM 25841, and carbadox on the dynamics of microbial composition in feces during the weaning transition period. The microbiota composition in different segments of the intestine were also investigated as ETEC F18 mainly target the small intestine of weaned pigs. Identifying shifts in microbial communities in the intestines could facilitate the development of nutritional interventions to control post-weaning diarrhea when the use of AGP is restricted, and to understand the impacts of currently available DFM on swine health.

### Fecal microbiota

Temporal analysis in fecal microbiota was performed using fecal samples collected throughout the experiment. Richness in microbial population was measured in Chao1 and richness and evenness were measured in Shannon index (Shannon, 1948; Chao, 1984, p. 1). Lack of significant treatment effect was observed in the Shannon diversity metric in fecal samples of pigs throughout the experiment. Microbial richness was increased during the habituation period (from day − 7 to day 0) but decreased from day 0 to day 7 PI in feces collected from pigs in the positive control, which indicate that ETEC F18 challenge reduced microbial richness in feces. Beta diversity measures the variability in microbial community composition among samples. Samples collected at the beginning of the experiment (day − 7) separately clustered from samples collected from other time points, suggesting weaning significantly shifted microbial community composition. Distinctive clusters were also observed between samples on day 0 and day 7 PI. However, feces collected from positive control were not fully separated from samples collected from negative control, which suggests the impacts of ETEC F18 on fecal microbial community composition might be limited. Results of alpha and beta diversities also suggest that dietary supplementation of antibiotics or *B. subtilis* have limited impacts on the microbial diversity in fecal samples.

In consistent with other published research (Frese et al., 2015; Li et al., 2020), Firmicutes and Bacteroidetes were the top two most abundant phyla in fecal samples of weaned pigs. As the age of pigs was increased, the relative abundance of Firmicutes were increased but the relative abundance of Bacteroidetes were decreased (Mach et al., 2015; Chen et al., 2017), indicating that the fecal microbiota has greatly shifted during post-weaning period. The likely reason was the sudden change in diet from sow milk to solid and plant-based ingredients and the changes in environment after weaning. Weaning and dietary change shift the swine core gut microbiome, including *Clostridiaceae1*, *Peptostreptococcaceae*, *Streptococcaceae*, *Bacteroidaceae*, *Enterobacteriaceae*, and *Lactobacillaceae* in feces (Mach et al., 2015; Chen et al., 2017; Luise et al., 2021). The increased *Lactobacillaceae* is highly correlated with enriched plant-derived mono- and di-saccharides (Frese et al., 2015). At the end of this experiment, most of pigs were recovered from weaning stress and ETEC infection as indicated by the absence of ETEC F18 in feces and normal diarrhea scores (He et al., 2020). The relative abundance of the core gut microbiota listed above was reversed in feces, suggesting that the impacts of weaning stress on gut microbiota are significant but temporary. When pigs adapt to their new diets and environment, diet becomes the main driver of regulating gut microbial composition.

Post-weaning ETEC infection can reduce pig appetite, disrupt intestinal barrier, and induce intestinal inflammation, all of which may contribute to the imbalance of the microbiota (Pollock et al., 2019). ETEC is categorized under the family of *Enterobacteriaceae*, which is likely the reason for the increased relative abundance of Proteobacteria and *Enterobacteriaceae* was observed in the current study. Similar results were also reported in mice inoculated with ETEC (Wang et al., 2018). Growing evidence suggests that an increased abundance of Proteobacteria might be associated with dysbiosis (Shin et al., 2015). ETEC infected pigs in the present study were fully recovered on day 21 PI, as reported no diarrhea was observed and no β-hemolytic coliforms were present in feces (He et al., 2020). Thus, the impacts of ETEC infection on fecal microbiome were also gradually reduced on day 21 PI.

The use of carbadox in feed also induced changes in the fecal microbiota. Carbadox is an oxidative DNA-damaging agent that mainly target gram-positive bacteria (Breijyeh et al., 2020). During habituation period, supplementation of carbadox reduced relative abundance of Firmicutes and *Lactobacillaceae* in feces. Although ETEC infection temporarily increased the relative abundance of *Lactobacillaceae* in feces of pigs in the antibiotics group, the abundance of *Lactobacillaceae* was sharply reduced when pigs were recovered from ETEC infection. The relative abundance of Bacteroidetes was also numerically higher in carbadox group than pigs in control. These observations indicate the impacts of in-feed antibiotics on gut microbiome were immediate (Lourenco et al., 2021). The reduced *Lactobacillaceae* in feces is consistent with previous research (Gao et al., 2018), suggesting that supplementation of carbadox modified the intestinal environment which may be not supportive to maintain the growth of some favorable bacteria in the intestines.

Compared with carbadox, the influences of *B. subtilis* on fecal microbiome of weaned pigs were limited throughout the experiment. The relative abundance of *Bacillaceae* was the greatest in feces fed with *B. subtilis* among all treatments on day 0 before ETEC infection and day 21 PI, which is likely due to *B. subtilis* is categorized under the family *Bacillaceae.* Supplementation of *B. subtilis* significantly enhanced the relative abundance of Firmicutes, but numerically reduced Proteobacteria and *Enterobacteriaceae* in feces at the peak of ETEC infection. This observation is also consistent with the published fecal culture results, in which pigs fed with *B. subtilis* had lower percentage of β-hemolytic coliforms in feces on day 7 PI (He et al., 2020). Our previous research revealed that both carbadox and *B. subtilis* supplementation were effective in enhancing growth performance and reducing diarrhea of weaned pigs challenged with ETEC F18 (He et al., 2020). However, results in fecal microbiome suggest that the impacts of dietary supplementation of *B. subtilis* and antibiotics on fecal microbiome were different in weaned pigs. The major highlights are the modulation of Firmicutes phylum. Overall, pigs fed with *B. subtilis* contained relative higher *Lactobacillaceae* but lower *Bacteroidaceae* than pigs fed with antibiotics throughout the experiment, although ETEC infection temporarily reduced this difference on day 7 PI. Although carbadox mainly targets gram-positive bacteria, it can also disturb bacterial DNA synthesis and induce the breakdown of chromosome in many gram-negative bacteria, including ETEC (Suter et al., 1978; Cheng et al., 2015). However, *B. subtilis* as a probiotic strain must colonize into the intestines of pigs in order to perform beneficial effects on gut ecosystem. Thus, it is not supersizing to observe the different impacts of these two supplements on fecal microbiome due to their different modes of action.

### Intestinal microbiota

In the current study, the intestinal microbiota changes were also analyzed in intestinal content collected at the end of experiment when pigs were fully recovered from ETEC infection. Similar to the results shown in Crespo-Piazuelo et al. (2018), Shannon and Chao1 index values in distal colon were higher than in jejunum and ileum. Consistently, beta diversity results showed clear separation between colon content vs. jejunal and ileal digesta, indicating the spatial heterogeneity of bacteria colonization across different intestinal sites (Mu et al., 2017). Supplementation of carbadox or *B. subtilis* had stronger influences on microbial diversity and composition in the ileum than in the jejunum and distal colon.

Ileal and jejunal digesta had greater relative abundance of Firmicutes and lowest relative abundance of Bacteroidetes than colon content, suggesting the luminal environment remarkably modulates digesta microbiome (Gao et al., 2018; Pollock et al., 2019). The relatively acidic environment and high oxygen level prevent the colonization of anaerobes in the small intestine (Donaldson et al., 2016). In addition, the small intestinal digesta contains more simple carbohydrates that could be utilized by the host and the microbiota, while the large intestine lumen comprises more complex carbohydrates for microbial fermentation (Zoetendal et al., 2012). The enriched *Lachnospiraceae*, *Ruminococcaceae*, and *Prevotellaceae* enable the large intestine’s capacity to degrade complex carbohydrates (Duncan et al., 2007; Arumugam et al., 2011; Zhang et al., 2018). The ileum is a major intestinal site where ETEC colonizes, which is probably the major reason that more abundant *Enterobacteriaceae* were observed in ileal digesta than jejunum and colon (Nagy et al., 1992). However, there were limited changes in the microbial composition in different intestinal segments when comparing positive control vs. negative control pigs on day 21 PI, which was likely due to the recovery of weaned pigs from ETEC F18 infection (He et al., 2020). The relative abundance of *Micrococcaceae* was proliferated in jejunal and ileal digesta of pigs infected with ETEC. *Micrococcaceae* are relatively abundant in newborn pigs, but are gradually reduced during pre-weaning period (Pena Cortes et al., 2018). The increased abundance of *Micrococcaceae* were also observed in diarrheal fecal samples in a human study (De et al., 2020). However, the reason for this change is not clear and needs to be further investigated.

In comparison to the positive control, carbadox supplementation had more influences on the intestinal microbiota than *B. subtilis*. In addition, carbadox supplementation altered jejunal and ileal microbiota more than colon microbiota in weaned pigs, which is in close agreement with a previous study using a mixed antibiotics supplementation (Mu et al., 2017). This observation also indicates that antibiotics treatment may mainly target the microbiota in the small intestine. At the taxonomic level, pigs fed with carbadox had greater relative abundance of gram-negative bacteria, including S*uccinivibrionaceae* and *Pasteurellaceae* in jejunum and *Muribaculaceae* in ileum, but lower relative abundance of *Lactobacillaceae* and *Bifidobacteriaceae* in ileum. This observation suggest that gram-negative bacteria may be more tolerant to carbadox due to their complicated outer membrane structure, containing lipopolysaccharides and peptidoglycans that can reduce the chance of carbadox penetrating into the bacterial membranes (Breijyeh et al., 2020). Gram-negative bacteria are more resistant to commonly used antibiotics by developing antibiotics resisting enzymes and undergoing mutations to increase resistance against antibiotics (Miller, 2016; Sumi et al., 2019). Therefore, the accumulation of gram-negative bacteria in the small intestine when carbadox is applied may increase the risk of developing antimicrobial resistance. However, in the production side, supplementation of carbadox was reported to effectively enhance growth performance and reduce intestinal inflammation of weaned pigs (Roof and Mahan, 1982; Stahly et al., 1997; He et al., 2020). The potential reason might include that carbadox can increase the population of short-chain fatty acid producing microbiota (i.e., *Ruminococcaceae*) in the small intestine, which may benefit to intestinal health and energy utilization in pigs (Bedford and Gong, 2018).

Pigs supplemented with *B. subtilis* had increased the relative abundance of gram-positive bacteria than pigs supplemented with carbadox, including *Lactobacillaceae, Bacillaceae*, and *Bifidobacteriaceae* in ileal digesta. The increased abundance of *Bacillaceae* was likely due to the colonization of *B. subtilis* in the intestinal tract (Pollock et al., 2019), which needs to be further confirmed in the future study. The more enriched *Lactobacillaceae* and *Bifidobacteriaceae* suggest that supplementing *B. subtilis* may help to maintain the desirable bacteria in the small intestine of weaned pigs, which may contribute to the concomitant decrease in enteric bacterial infection. Previous research also reported that *B. subtilis* supplementation can also decrease luminal pH and oxidation potential of the gut matrix, which may provide a favorable condition for *Lactobacillaceae* to thrive but inhibit the growth of the pathogenic bacteria (Yang et al., 2015). Therefore, results from the current study indicate that the modulation of gut microbiome by *B. subtilis* supplementation also contributes to the reduced diarrhea and enhanced intestinal health of ETEC infected pigs (He et al., 2020).

## Conclusion

Our previous study reported that supplementation of *B. subtilis* DSM 25841 improved growth performance and disease resistance of weaned pigs under ETEC challenge (He et al., 2020). The current study characterized the microbial diversity and composition of fecal samples and intestinal contents collected from He et al. (2020). The present results indicate that weaning stress, age of pigs, ETEC infection, and dietary supplements all contributed to the fecal and intestinal microbiome changes in weaned pigs. Supplementation of carbadox and *B. subtilis* differently modified fecal and intestinal microbiota. Pigs supplemented with *B. subtilis* had higher abundance of *Bacillaceae* than pigs supplemented with carbadox in fecal and intestinal content. Supplementation of carbadox increased the relative abundance of gram-negative bacteria including Bacteroidetes and Proteobacteria which may impose risk of increasing antibiotics resistance. Supplementation of *B. subtilis* was associated with an increase or maintenance of the relative abundance of beneficial bacteria including *Lactobacillaceae* and *Bifidobacteriaceae* in the ileum and colon. Future research is needed to quantify *B. subtilis* colonization in the gastrointestinal tract and to investigate the interactions of *B. subtilis* with other bacteria, especially ETEC. In addition, the limits of 16S rRNA sequencing has been recognized, thus, the analysis of gut bacterial biomass and the functional genomes from the gut microbiota should be considered in the future research as well.

## Data availability statement

The datasets presented in this study can be found in online repositories. The names of the repository/repositories and accession number(s) can be found at: https://www.ncbi.nlm.nih.gov/, PRJNA885119.

## Ethics statement

Animal procedures were reviewed and approved by the Institutional Animal Care and Use Committee (IACUC #19322) at the University of California, Davis (UC Davis).

## Author contributions

YL and XL designed and supervised the entire experiment. CJ performed all gut microbiome analysis, including sample analysis and data analysis. CJ wrote the manuscript. YL and XL revised and approved the final version. All authors contributed to the article and approved the submitted version.

## Conflict of interest

The authors declare that the research was conducted in the absence of any commercial or financial relationships that could be construed as a potential conflict of interest.

## Publisher’s note

All claims expressed in this article are solely those of the authors and do not necessarily represent those of their affiliated organizations, or those of the publisher, the editors and the reviewers. Any product that may be evaluated in this article, or claim that may be made by its manufacturer, is not guaranteed or endorsed by the publisher.

## Footnotes

^1^ https://github.com/najoshi/sabre
